# Enhanced and long term immunogenicity of a Her-2/neu multi-epitope vaccine conjugated to the carrier CRM197 in conjunction with the adjuvant Montanide

**DOI:** 10.1186/s12885-017-3098-7

**Published:** 2017-02-09

**Authors:** Joshua Tobias, Joanna Jasinska, Karin Baier, Michael Kundi, Nicholas Ede, Christoph Zielinski, Ursula Wiedermann

**Affiliations:** 1 Institute of Specific Prophylaxis and Tropical Medicine, Center for Pathophysiology, Infectiology and Immunology, Medical University of Vienna, Kinderspitalgasse 15, 1090 Vienna, Austria; 2 Institute of Environmental Health, Medical University of Vienna, 1090 Vienna, Austria; 3 Imugene Ltd., Suite 1, 1233 High Street, Armadale, Melbourne, VIC 3143 Australia; 4 Division of Oncology, Department of Medicine I, Medical University of Vienna, General Hospital, Vienna, Austria

**Keywords:** Her-2/neu, Hybrid peptide, Adjuvant, Mice, Humoral and cellular response, Th1-deriving response

## Abstract

**Background:**

We previously identified three short single peptides (P4, P6 and P7) representing different B-cell epitopes on the extracellular domain of Her-2/neu for a vaccine that was tested in a phase-I clinical trial. Here we describe the improvement of the multi peptide vaccine by fusing the single peptides to a hybrid peptide P467.

**Methods:**

After coupling to either virosomes or to diphtheria toxoid CRM197 (CRM), the hybrid peptide was tested in different concentrations in combination with either Montanide or Aluminium hydroxide (Alum) in preclinical studies.

**Results:**

Already low amount (10 μg) of P467 conjugated to CRM led to faster onset of high antibody levels compared to the P467-virosome. The formulation P467-CRM-Montanide induced higher serum IgG antibody titers, compared with P467-CRM-Alum, as examined by ELISA using recombinant Her-2/neu or Her-2/neu natively expressed on the tumor cell line SK-BR-3. Compared to P467-CRM-Alum, higher in vitro production of IL-2 and IFNγ in the Montanide-immunized mice was induced after re-stimulation of splenocytes with CRM but also with P467, indicating a clear Th1-biased response. In contrast to the single B cell peptides, the hybrid peptide led to T cell proliferation and cytokine production as CD4 T cell epitopes were generated in the fusion region of the single peptides P4 and P6 or P6 and P7. Additionally, a significantly higher proportion IFNγ-producing CD8+ T cells was found in the P467-CRM-Montanide immunized mice, probably by Montanide-driven bystander activation. Importantly, anti-P467 IgG antibodies exhibited anti-tumor properties and the combination of anti-P467 specific IgG with Herceptin® was found to inhibit the proliferation of Her-2/neu-overexpressing cell line SK-BR-3 in a significantly higher capacity than Herceptin® alone.

**Conclusions:**

Fusion of the B cell peptides has led to additional generation of CD4 T cell epitopes, and this P467-multi epitope vaccine was found to induce polyclonal antibody responses with anti-proliferative capacity against Her-2/neu. The hybrid vaccine together with Montanide induced higher and long-lasting antibody levels, Th1-biased cellular responses being superior to vaccination with the single B cell peptides. This vaccine formulation is now planned to be evaluated in a phase Ib/II study in Her-2/neu overexpressing cancer patients.

## Background

The 185 kDa transmembrane receptor oncoprotein Her-2/neu, known also as ErbB-2, is a member of the ErbB/epidermal growth factor receptors and class I family of receptor tyrosine kinases [1]. The oncoprotein has been shown to be involved in tumor progression, over- expressed on tumor cells of e.g. breast and gastric cancers, and also associated with poor disease outcome [2]. Therefore Her-2/neu represents an excellent target for the development of therapeutic agents [3].

Peptide-based vaccines offer several advantages, including focusing the immune response to relevant epitopes and avoiding non-protective responses, potentiating their use for cancer immune-therapy [4, 5]. In line with this approach, our group has earlier identified three single peptides with B cell peptides located in different regions of the extracellular domains of Her-2/neu, i.e. P4, P6 and P7 [6]. We demonstrated that immunization with the single peptides led to induction of antibodies with the capacity to inhibit tumor-growth in vitro, as shown by proliferation assays, complement dependent- and antibody dependent-cell cytotoxicity assays [6]. In a breast cancer mouse model with activated c-*neu* oncogene, we further demonstrated that immunization with the mixture of the three peptides each coupled to tetanus toxoid elicited anti-tumor efficacy. Co-application of the vaccine with IL-12 was associated with a Th1-polarized immune response which demonstrated elevated Her-2/neu-specific IgG levels and increased in vitro production of IFNγ by splenocytes [7].

Virosomes, with an intrinsic adjuvant activity, support antibody formation and induction of T-helper cell responses against surface-associated antigens and have been used in human vaccines against e.g. influenza or hepatitis A [8, 9] showing strong immunogenicity. Accordingly, for clinical use, our multi-peptide vaccine containing the single peptides conjugated to virosomes was examined in a phase I study with breast cancer patients in end stage of the disease [10]. While the study showed good immunogenicity as well as an excellent safety profile [10], several drawbacks of the virosomal formulations including solubility and limited stability after coupling all the single peptides together to virosomes, were the reasons to reconstruct and improve the multi-peptide vaccine with respect to specificity and clinical applicability. One possibility was to fuse the three single peptides into one long hybrid peptide [11]. Different combinatorial orders of the single peptides were therefore constructed and tested [12], eliciting two candidate hybrid peptides as potentially immunogenic, designated as P467 and P647.

The carrier protein CRM197 (CRM; Cross Linking Materials) is an enzymatically inactive and nontoxic [toxoid] form of diphtheria toxin [13], and has been successfully used in many vaccines against infectious diseases [14]. CRM rapidly activates CD4+ T cells with a heterogeneous Th1 and Th2 cytokine profile for activating B cells and regulating the quantity of the induced antibodies [15], and therefore provides an alternative conjugation partner for the peptides over virosomes. Additionally, the use of adjuvants with Th1-promoting properties has been shown to be of importance to enhance antitumor effects and reduce vascularization within various tumor microenvironments [16, 17]. The aim of the current study was therefore to compare the immunogenicity of the selected hybrid peptide in mice, 1) when coupled to CRM compared to virosomes to select a potent carrier for the hybrid peptide vaccine, and 2) when administered together with Montanide (a Th1 driving adjuvant, with capacity to induce both antibody and cellular responses) [18] or Alum (a Th2 driving adjuvant) [19] to select an adjuvant which gives more potent immune responses with anti-tumor effects. Our results show that the peptide conjugated to CRM promotes induction of antibody responses, and in addition to humoral responses also cellular responses are induced at significantly higher levels with lower amounts of the peptide conjugate when administered together with Montanide in contrast to Alum.

## Methods

### Peptides

For immunization studies, the single peptides P4 (PESFDGDPASNTAPLQP), P6 (RVLQGLPREYVNARHC) and P7 (YMPIWKFPDEEGAC) [6, 7] were used to construct the hybrid peptides P467 (PESFDGDPASNTAPLQPRVLQGLPREYVNARHSLPYMPIWKFPDEEGAC) and P647 (RVLQGLPREYVNARHSPESFDGDPASNTAPLQPYMPIWKFPDEEGAC) which were designed at Pevion (Switzerland) and synthetized at Bachem (Switzerland); during the synthesis of P467 and P647, the Cysteine (C) of P6 was replaced by ‘SLP’ or ‘S’, respectively, as underlined. In the immunization experiment-I both hybrid peptides were coupled to either virosomes or to CRM (Mymetics, The Netherlands), and in experiment-II the hybrid peptide was only coupled to CRM (piCHEM, Austria). Of importance to indicate that after conjugations, the P647-CRM conjugate precipitated whereas P467-CRM remained soluble in water-based buffer. To evaluate antibody responses directed to the fusion peptides, non-conjugated P467 and P647 were used as coating antigen.

For T cell epitope mapping, a panel of 20 overlapping peptides spanning the entire sequence of the hybrid peptide P467, each with 12 aa length and offset of 2 aa, as well as each of the single peptides (Cambridge Research Biochemicals Limited, Cambridge, UK), were used.

### Adjuvants

The hybrid peptides P467 and P647, conjugated to either CRM or virosomes, were examined with two different adjuvants, namely Alum (Aluminium hydroxide; Brenntag, Denmark/ Serva, Germany), or Montanide ISA-51-VG (Seppic, France) which is a water-in-oil emulsion. The amounts of the adjuvants were calculated based on the amounts of the peptides administered in the immunizations. The mixing of P467-CRM with Montanide, using 2-syringe mixing method, or Alum adjuvants was carried out according to the manufacturers’ instructions. The adjuvants, mixed with NaCl (Montanide) or PBS (Alum), were used as controls.

### Mice immunizations

**Fig. 1:**
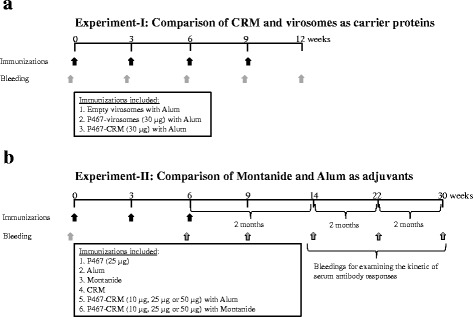
Experimental design. Female Balb/C mice were immunized with different amounts of P467 and P647 hybrid peptides, conjugated to either CRM or virosomes (1**a**; Experiment-I), or with different amounts of P467-CRM administered together with Alum or Montanide (1**b**; Experiment-II). For examining the kinetic of the immune responses, additional blood samples were taken in two months intervals for the period of 6 months after the last immunization. The occasions for immunization (*black arrows*) and bleeding (*grey arrows*) are indicated

Female Balb/C mice (Charles River, Sulzfeld, Germany; 6–8 week of age at the time of delivery) were used in two subcutaneous immunization studies, i.e. experiment-I (Fig. 1a) followed by experiment-II (Fig. 1b). The experiments were approved by the Animal Experimentation Committee of the Medical University of Vienna and the University of Veterinary Medicine as well as by the Austrian Federal Ministry of Science and Research (BMWF-66.009/0203-WF/V/3b/2015).

In experiment-I (5 mice per group), aiming to compare virosomes and CRM as carriers, the immunizations with conjugated constructs started 16 days after the priming. All virosomal formulations were delivered ready to use and were applied without any additives. CRM-conjugates stocks were diluted with NaCl solution and mixed with Alum prior injection. Of note, that P647 conjugated to CRM precipitated while no precipitation of the P467-CRM conjugate was observed. CRM-P647 was vortexed and the suspension was used in the same manner as CRM-P467. Four immunizations were given in 3 weeks intervals, and blood samples were taken prior each immunization and three weeks after the last immunization when the mice were sacrificed.

In the follow up experiment-II (8 mice per group), aiming to compare Alum and Montanide as adjuvants, three immunizations were given in 3 weeks intervals, and blood samples were taken prior the first and third immunization, as well as three weeks after the last immunization. In addition, for examining the kinetic of the immune responses, additional blood samples were taken in two months intervals for the period of 6 months after the last immunization.

### Detection of peptide-specific serum IgG

Microtiter plates (Nunc Maxisorp, Denmark) were coated with uncoupled peptides P467 or P647 (Bachem, Switzerland), in carbonate buffer (0.5 μg/well), and ELISA was performed as previously described [6]. After blocking, diluted sera from the immunized mice were added. Bound IgG were detected with HRP-labelled rabbit anti mouse IgG antibody and subsequent TMB staining. For detection of IgG1 and IgG2a isotypes, rat anti mouse IgG1 or rat anti mouse IgG2a (BD Biosciences, USA) and the secondary antibody HRP-labelled mouse anti-rat IgG (Jackson Immuno Research, USA) were used, followed by TMB staining. Plates were read after adding stop solution at 450 vs 630 nm.

### Detection of Her-2/neu-specific IgG

A fusion protein consisting of the recombinant extracellular domain of human Her- 2/neu (aa 23–652) fused to Fc region of human IgG1 (ErbB2/Fc Chimera, R&D Systems) was used as coating antigen. Plates were coated with 0,1 μg/well, and detection of Her-2/neu specific IgG antibodies as well as subclasses IgG1 and IgG2a was carried out as described above.

### Cytokines production in cultures of splenocytes

Mice (*n* = 4/group) immunized with P467-CRM construct and administered with Alum or Montanide (experiment-II), were sacrificed 18 days after the third immunization. Splenocytes were taken aseptically, minced, sterile-filtered and cell suspensions were prepared as previously described [20]. Cells (5 × 10^5^ per well) were plated in 96-well round-bottomed plates, and stimulated with medium alone, CRM, or unconjugated P467 at concentration of 20 μg/ml for 72 h in culture medium (RPMI 1640, with 10% heat- inactivated FCS, 2 mM L-Glutamine) at 37 °C, 95% humidity and 5% CO_2._ Supernatants were harvested and stored at −20 °C, until analysis. Levels of secreted IL-2, IFNγ and IL-5 were measured by ELISA according to manufacturer’s instructions (Affymetrix eBioscience, USA), and expressed in pg/ml.

### Surface marker staining of lymphocyte populations by FACS analysis of splenocytes

Freshly isolated splenocytes (1 × 10^6^ cells) were stained for characterization of lymphocytes sub-populations, i.e. T cells (CD3 + CD4+ and CD3 + CD8+), B cells (CD3-CD19+) and NK cells (CD3-CD335+), using fluorochrome-conjugated antibodies CD3 FITC, CD4 PerCP, CD8a (Ly-2) PE, CD19 APCeFluor780 and CD335 (NKp46) eFluor450 (eBioscience). Pooled samples (per group) were used as negative and FMO (Fluorescence Minus One) controls in addition to Isotype controls.

### Surface staining of Her-2/neu overexpressing cell line by FACS analysis

The Her-2/neu-overexpressing human breast cancer cell line SK-BR-3 and the human melanoma cell line 518.A2 as control cells (Her-2/neu negative) [6] were used to evaluate the binding capacity of the sera raised against the P467-CRM-Alum or P467-CRM-Montanide (experiment-II). Cells were cultivated as described previously [6], resuspended in FACS buffer and blocked with human Ab serum. Antibody binding of the examined mice sera, and Herceptin® (a humanized IgG1 antibody), were detected with PE-conjugated secondary F(ab’)2 Anti-mouse IgG and Anti-Human IgG (Fc gamma-specific) (eBioscience), respectively.

### Intracellular staining of IFNγ production by single cell analysis

Freshly isolated splenocytes (2,5 × 10^6^ cells/ml in 24 flat bottom well plate) were stimulated for 2 h at 37 °C, with PMA (Sigma; Phorbol Myristate Acetate, 10 ng/ml) and Ionomycin (Sigma; 1,25 μM), and additional 4 h with Brefeldin A (Sigma; 10 μg/ml) to avoid secretion of cytokines. Cells were then split into micronic tubes (1 × 10^6^/cells per tube), blocked with Fc Block (anti-mouse CD16/32) and used for surface staining of CD3, CD4, CD8, CD19 and CD335, using the above mentioned fluorochrome-conjugated antibodies. Cells were then fixed and permeabilized, followed by intracellular staining with IFNγ using fluorochrome-conjugated antibodies IFNγMaB APC, and acquired on FACS Canto and analyzed with CellQuestPro Software (BD).

### T cell epitope mapping

Splenocytes from the mice immunized with 25 μg of P467-CRM with Montanide (i.e. experiment-II) were prepared as mentioned above, added into 96-well round-bottomed plates (2 × 10^5^ per well) and re-stimulated with the single peptides P4, P6 and P7, the hybrid peptide P467 or each of the over-lapping peptides of P467 (2 μg/well), for 96 h. ConA stimulation was used as positive control. To determine T cell proliferation, the stimulated cells in each well were pulsed with 0.5 μCi [^3^H] thymidine (Perkin Elemer) for the last 16–18 h of stimulation and incorporated ^3^H was measured using a liquid scintillation counter for measuring the CPM (counts per minute). The proliferative responses were expressed as stimulation index (SI), where SI = CPM for test culture divided by CPM for unstimulated cells (baseline level). SI ≥ 2 was considered as the capacity of the examined peptides to stimulate T cells.

### Tumor cell proliferation inhibition assay

To examine the inhibitory capacity of P467-specific antibodies on proliferation of the Her-2/neu-overexpressing cell line SK-BR-3, or the cell line 518.A2 as control cells, New Zeeland White rabbits were immunized according to the protocol of the laboratory of Charles River (Châtillon-sur-Chalaronne, France) and serum IgG antibodies were isolated as described previously [7]. The capacity of the purified P467-specific antibodies, Herceptin® alone, or a combination of both, with 10 μg of each examined antibody, was evaluated in vitro using [3H]-thymidine proliferation assay as described above.

### Statistical analyses

Longitudinal data were analyzed by mixed ANOVA models with repeated measures and two group factors (dosage and type of adjuvant). For grouped data two-factor (dose and adjuvant) ANOVA was performed. Comparison of each time point of dose level was performed by linear contrasts. For IFNγ producing T-cells the comparison of the two adjuvants was done by Student’s t-tests with two-tailed *p*-values considered. ANOVA and linear contrasts of arcsine transformed data were applied for analysis of the proliferation inhibition results. Significant differences were indicated as *P* values <0.05 (*), 0.001–0.01 (**) or <0.001 (***).

## Results

### CRM-conjugated hybrid peptides are superior over virosomal conjugates in inducing high antibody responses already after two vaccine administrations

**Fig. 2:**
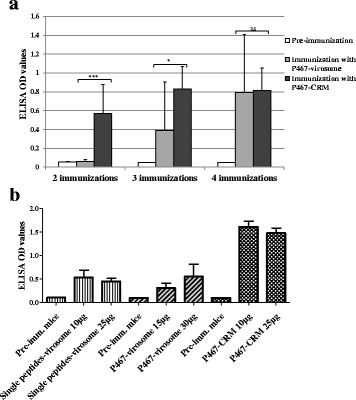
Level of serum IgG antibody responses against recombinant extracellular Her-2/neu. **a** Kinetic of the antibody responses induced after 2, 3, and 4 immunizations with 30 μg of the hybrid peptide P467 conjugated to virosomes or CRM, are shown. **b** Comparison of the levels of the antibody responses induced after 3 immunizations with virosomal constructs expressing a mixture of single peptides (10 μg or 25 μg), virosomal constructs expressing the P467 peptide (15 μg or 30 μg), or the CRM-conjugated P467 administered together with Montanide (10 μg or 25 μg) is shown. The OD values are based on ELISA experiments as described in materials and methods. The statistical analysis was performed using ANOVA (two-way) and linear contrasts. Significant differences are denoted by *asterisks*

To check the number of administered doses required for induction of strongest and quickest antibody responses against the hybrid peptides P467 or P647, mice were first immunized with 30 μg of each peptide, conjugated to either CRM or virosomes (Fig. 1a; experiment-I). Already after administration of 2 doses, the titers of serum IgG antibody against recombinant Her-2 were shown to be significantly higher in mice immunized with P467-CRM conjugate than with P467-virosomes (Fig. 2a). After 4 immunizations both P467-conjugated formulations induced comparable and high levels of serum IgG responses (Fig. 2a). In an additional examination we further compared the titers of IgG antibodies in sera of mice previously immunized in three occasions with the mixture of single peptides P4, P6 and P7 expressed on virosomes, or the hybrid peptide P467 expressed on virosomes, or with CRM-conjugated hybrid peptide P467 administered together with adjuvant (Montanide) (Fig. 2b). While no difference between single peptides and the hybrid peptide with regards to their immunogenicity was observed, the immunogenicity of the CRM-conjugated hybrid peptide was shown to be markedly superior over virosomes (Fig. 2b). These results all together indicated the superiority of CRM as a carrier protein over virosomes, leading to not only to more rapidly induced but also to higher antibody titers with binding capacity to the recombinant extracellular Her-2/neu. The hybrid peptide P647 conjugated to CRM, but not to virosomes, induced similar higher immune responses as P467-CRM conjugate already after two rounds of immunizations (data not shown). However due to problems with solubility of the P647 hybrid peptide it was decided to focus on P467 and evaluate its immunogenicity in mice in a follow up immunization (Fig. 1b; experiment-II) in which the adjuvants Montanide and Alum were examined and compared.

### A lower dose of P467-CRM administered with Montanide, compared to Alum, induces higher antibody responses along with mixed Th1/Th2 profile

**Fig. 3:**
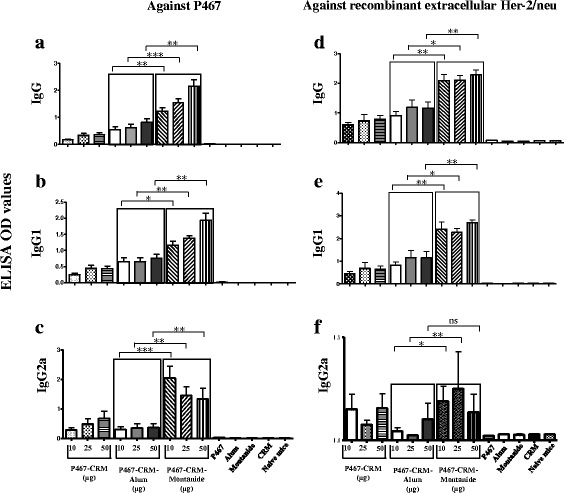
Levels of serum IgG, IgG1 and IgG2a antibody responses against P467 and recombinant extracellular Her-2/neu. The levels of serum IgG, IgG1 and IgG2a against P467 (3**a**, 3**b** and 3**c**) or recombinant extracellular Her-2/neu (3**d**, 3**e** and 3**f**), induced after 3 immunizations with different concentrations of the conjugate administered with Alum or Montanide, are shown. The OD values are based on ELISA experiments as described in materials and methods. The statistical analysis was performed using ANOVA (two-way) and linear contrasts. Significant differences are denoted by *asterisks*. ns, not significant

To evaluate the antibody responses induced by the P467-CRM, mice were immunized with 10, 25 or 50 μg of the conjugate, administered with either Alum or Montanide. Three weeks after the last immunization, the lowest dose of the conjugate (10 μg) was shown to induce significantly higher antibody levels of IgG (Fig. 3a), IgG1 (Fig. 3b) and IgG2a (Fig. 3c) against P467 when administered with Montanide, compared to mice administered with Alum or sham-induced control groups. Her-2/neu specific IgG (Fig. 3d) and IgG1 (Fig. 3e) antibody responses were also significantly higher in Montanide than Alum-immunized mice. The anti-Her-2/neu IgG2a antibody titers in mice immunized with 10 μg and 25 μg of the P467-CRM and Montanide were also significantly higher than when compared to Alum (Fig. 3f). These results indicated that administration of only 10 μg of the P467-CRM conjugate with Montanide can induce significantly higher Th1-polarized antibody responses than the higher dose (50 μg) of the peptide-conjugate administered with Alum.

To further evaluate if the antibodies induced by P467-CRM-Montanide or with P467-CRM-Alum bind to native Her-2/neu, sera of the immunized mice were used for staining of Her-2/neu overexpressing breast cancer cell line SK-BR-3 or the human melanoma cell line 518.A2 as control cells (Her-2/neu negative), and analyzed by FACS. Considerably higher number of SK-BR-3 cells was detected in case of sera raised against P467-CRM-Montanide, when compared to P467-CRM-Alum (Table 1). Marginal to undetectable number of SK-BR-3 cells was stained with the sera of mice before the immunizations, or when the control cells 518.A2 were examined with sera against either the formulations, indicating the binding specificity of the antibodies (Table 1). These results not only show the higher antibody titers induced by the sera against P467-CRM-Montanide, as observed by ELISA assay, but also their similar binding capacity to the recombinantly produced Her-2/neu and to the Her2/neu natively expressed on the SK-BR-3 cells.

**Table 1:** Binding mice sera, immunized with P467-CRM-Alum or P467-CRM-Montanide, to positive or negative Her-2/neu-overexpressing cell lines

	Percentage of detected examined cells^a^	
Sera of mice before, and after immunization with:	SK-BR-3	518.A2
Pre-immunization	2.35	1.75
P467-CRM-Alum (10 μg)	16	3.3
P467-CRM-Alum (25 μg)	6.9	1.8
P467-CRM-Alum (50 μg)	5.6	1.4
P467-CRM-Montanide (10 μg)	21.6	6.2
P467-CRM-Montanide (25 μg)	46.4	3.7
P467-CRM-Montanide (50 μg)	17.6	2.4

^a^The values indicate the percentage of the stained Her-2/neu overexpressing SK-BR-3 cells and 518.A2 cells as control cells (Her-2/neu negative) cells, detected by FACS analysis, as described in material and methods. The sera were diluted 1/100

**Fig. 4:**
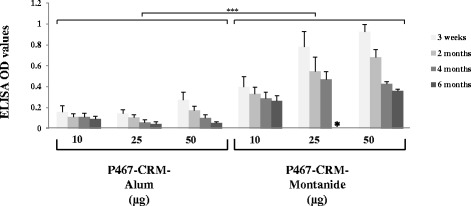
Kinetic of serum IgG antibody responses against P467. The levels of serum IgG antibody responses, induced 2, 4 and 6 months after the last immunization with different amounts of P467-CRM conjugate administered with Alum or Montanide, are shown. * The mice in this group were sacrificed before the six-month period, and their splenocytes were used for the T cell epitope mapping analysis (Table 2). The OD values are after subtraction of the background (i.e. pre-immunized mice), and based on ELISA experiments as described in materials and methods. The statistical analysis was performed using Generalized Estimation Equations. Significant difference is denoted by ***

The kinetic of the serum IgG responses for a period of six months after the last immunization was also examined. Compared to Alum, at all examined time points and during the course of the examined period, significantly higher IgG responses against P467 in mice administered with Montanide were observed (Fig. 4). Accordingly, also higher Her-2/neu specific IgG responses were measured (data not shown). Although no significant difference between the decline rate of the responses was found (data not shown), the results indicate the long term immunogenicity of the P467-CRM-Montanide formulation.

### Immunization with P467-CRM administered with Montanide, compared to Alum, induces higher in vitro production of IL-2, IFNγ and IL-5 upon re-stimulation with CRM and P467

**Fig. 5:**
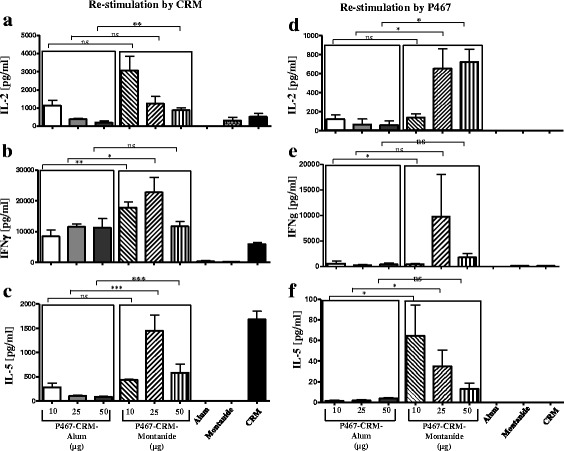
Levels of cytokines, IL-2, IFNγ and IL-5, produced in vitro. Levels of IL-2, IFNγ and IL-5 produced in vitro in splenocytes of mice immunized with different amounts of P467-CRM conjugate administered with Alum or Montanide, after re-stimulation with CRM (5**a**, 5**b** and 5**c**) or P467 (5**d**, 5**e** and 5**f**), are shown. Splenocytes of the immunized mice were used and re-stimulated, as described in materials and methods. The statistical analysis was performed using ANOVA (two-way) and linear contrasts. The measured levels of the controls (Alum Montanide and CRM) in Fig. 5d and f were lower than the background, and therefore were set to zero. Significant differences are denoted by *asterisks*. ns, not significant

Splenocyte cultures from mice immunized with P467-CRM together with Alum or Montanide were re-stimulated and examined for in vitro cytokines production. Production of IL-2, IFNγ and IL-5 (Fig. 5a, b and c, respectively) was shown to be significantly higher after stimulation with CRM in mice administered with Montanide, compared to Alum. Interestingly, the cytokine production was also observed upon re-stimulation of the splenocytes with the hybrid peptide P467 (Fig. 5d, e and f), indicating formation of a T cell epitope after fusion of the single B cell epitopes peptides in P467 with capacity to stimulate splenocytes.

### T-cell epitope-mapping of P467

**Fig. 6:**
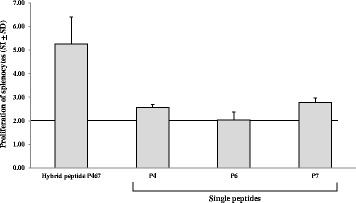
T cell stimulation capacity of the hybrid peptide P467 and single B cell epitope peptides. Splenocytes of mice immunized with 25 μg of P467-CRM together Montanide were re-stimulated with the hybrid peptide P467 or the single B cell epitope peptides P4, P6 and P7, as described in materials and methods. The *bars* indicate stimulation indexes (SI) in the presence of each examined peptide. The *horizontal line* indicates the cut-off of 2.0, above which the calculated values of SI were considered as significant

Based on the observation that the hybrid peptide P467 has induced in vitro production of cytokines after re-stimulation of splenocytes (Fig. 5d–f), splenocytes of mice which had been immunized with 25 μg of P467-CRM plus Montanide were re-stimulated with P467 or individually with each of the single peptides P4, P6 or P7. Comparing the stimulation index (SI) values, the hybrid peptide led to marked stimulation of splenocytes as compared to the single (B cell) peptides (Fig. 6). To identify the T cell immunodominant regions in P467, epitope mapping was carried out by re-stimulating splenocytes of mice which had been immunized with 25 μg of P467-CRM plus Montanide, with series of overlapping peptides spanning the entire sequence of P467. Comparing the SI values (SI > 2), three overlapping peptides were identified as potential immunodominant CD4 T cell epitopes: NTAPLQPRVLQG, SLPYMPIWKFPD and MPIWKFPDEEG, corresponding to the aa 11–22, 33–44, 37–47 of P467, respectively, were identified (Table 2). These results clearly indicated that the fusion of the single peptides has generated novel T cell epitopes in the hybrid peptide with T cell proliferative capacity of the hybrid peptide in contrast to the single B cell epitope peptides.

**Table 2:**
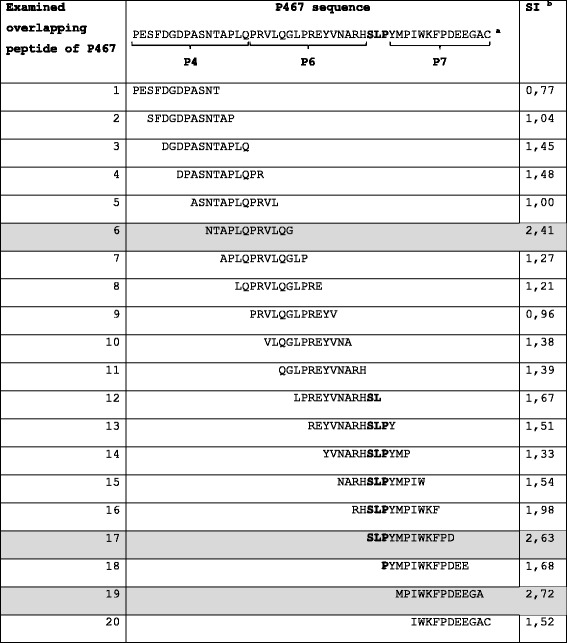
T cell epitope mapping of the hybrid peptide P467

^a^The positions of the single B cell epitope peptides P4, P6 and P7 are indicated under the sequence of the hybrid peptide P467. The amino acids SLP, which were added during the fusion of P6 and P7, are shown in bold

^b^Values indicate the stimulation indexes measured after stimulation of mice splenocytes with the examined overlapping peptides, as described in materials and methods, and are representative of two sets of experiments. The overlapping peptides with SI above 2 are highlighted

### Montanide propagates expansion of IFNγ-producing CD8 T cell

**Fig. 7:**
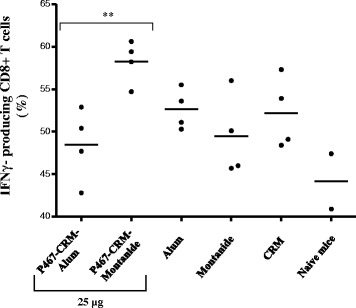
Level of INFγ-producing CD8+ T cells. The proportions of INFγ − producing CD8+ T cells in mice immunized with 25 μg of P467-CRM conjugate administered with Alum or Montanide, are shown. The statistical analysis was performed using *t* test. Significant difference is denoted by *asterisks*

The capacity of Montanide versus Alum in expanding lymphocytes’ subpopulations, in particular IFNγ producing CD4 and CD8 T cells, was further evaluated by FACS analyses on splenocytes of mice sacrificed three weeks after the last immunization. The results indicated expansion of T cells, of both CD4, CD8, as well as B cells and NK cells in all examined groups (data not shown). Since immunization with 25 μg of the hybrid peptide plus Montanide was associated with the higher cytokine responses, we further evaluated at single cell level the IFNγ production by splenocytes of mice immunized with 25 μg of P467-CRM administered with either Alum or Montanide. The results showed IFNγ-producing CD8 T cells in significantly higher levels especially in mice administered with Montanide rather than with Alum (Fig. 7). IFNγ production by CD4 T cells and NK cells did not differ between the groups (data not shown). These results indicated enhanced capacity of Montanide versus Alum to propagate the expansion of different splenocyte subclasses, particularly of IFNγ-production CD8 T cells.

### Anti-tumor activity of induced antibodies

**Fig. 8:**
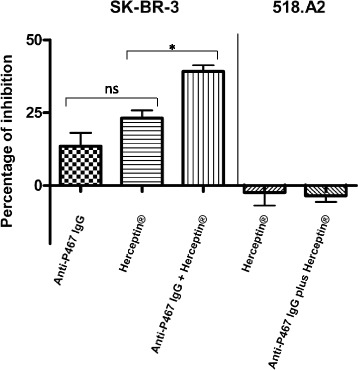
Proliferation inhibition of SK-BR-3 cells in vitro. [3H]-thymidine proliferation assay demonstrating the effects of purified anti-P467 rabbit IgG antibodies on the Her-2/neu-overexpressing human breast cancer cell line SK-BR-3 and the human melanoma cell line 518.A2 as control cells (Her-2/neu negative). Data are expressed in percentage of inhibition, based on the mean (+ SEM) of the tested antibody isolates; cpm values of untreated wells were set to 100%. Significant difference is denoted by *. ns, not significant

To assess the proliferation (tumor growth) inhibition capacity of anti-P467 antibodies induced in rabbits, purified P467-specific IgG antibodies were tested in vitro using [3H]-thymidine proliferation assay with Her-2/neu overexpressing SK-BR-3 cells. Incubation of SK-BR-3 cells with P467-IgG antibodies led to reduction of tumor cells proliferation compared to untreated SK-BR-3 cells, which was more pronounced (not significant) when cells were incubated with Herceptin® (Fig. 8). However, combining the examined purified IgG antibodies with Herceptin® showed a significantly higher proliferation inhibition than Herceptin® alone (Fig. 8), indicating the additive effect of the P467-specific IgGs to Herceptin® in inhibiting the growth of the examined Her-2/neu overexpressing tumor cells. Neither Herceptin® nor the anti-P467 antibodies in combination with Herceptin® led to reduction of tumor growth of the Her-2/neu negative tumor cell line (518.A2), showing the specificity of the anti-tumor activities of the tested antibodies.

## Discussion

We previously demonstrated that the 3 selected B cell peptides of Her-2/neu, P4, P6 and P7, conjugated to immune-stimulatory virosomes gave rise to Her-2/neu specific antibodies with antitumor activity [6, 7]. Despite an excellent safety and immunogenicity profile tested in a phase-I study [10], problems of solubility and limited stability of the formulation after coupling of the single peptides to virosomes, were the reasons to improve the formulation of the multi-peptide vaccine. Therefore, the three single peptides were fused to each other to a long hybrid peptide, and in this study the immunogenicity of the hybrid peptide was investigated.

The feasibility of the mutated diphtheria toxoid CRM as a carrier protein, and as an alternative to virosomes, was first tested after conjugation with the two examined hybrid peptides containing the single peptides fused in different combinatorial orders, i.e. P467 and P647. Already after 2 immunizations, the CRM-conjugated hybrid peptides were shown to induce significantly higher serum antibody responses against Her-2/neu compared to the virosome-based formulations. We further showed similar capacity of the single peptides and the hybrid peptide when expressed on virosomes in inducing anti-Her-2/neu serum antibody responses, and CRM-conjugation of the hybrid peptide was shown to markedly increase the antibody titers indicating the superiority of the CRM rather than virosomes as carrier protein for conjugation of the hybrid peptide. The rapid antibody responses shown by the CRM-conjugates may be of importance in clinical settings as additional to chemotherapy effects, and early onset of immune responses is important for translation into antitumor responses. This further signifies the advantage of CRM over the virosome as a suitable carrier protein for our hybrid peptides. Based on the observed rapid immune responses with CRM, and precipitation issues with the hybrid peptide P647, the P467-CRM conjugate was chosen for further investigation in a standard immunization regimen (Fig. 1b; Experiment II,).

In addition to humoral responses induced by the hybrid peptide P467, in vitro re-stimulation of splenocytes with the peptide induced high levels of IL-2, IFNγ and IL-5 production. This is of particular interest, as the single peptides P4, P6 and P7 had been identified as B cell epitopes with no capacity to stimulate T cells, suggesting that fusion of the single peptides has formed epitopes recognized by T cells. This was verified by examining splenocytes, re-stimulated by series of short (12 aa) overlapping peptides spanning the entire sequence of P467 or each of the single peptides. Indeed, the hybrid peptide led to nearly 3-fold higher stimulation index than the single peptides, and the immunodominant T cell epitopes were shown to be located at the regions where the single peptides are fused. The third immunodominant CD4 T cell epitope is within the single peptide P7. Since the single peptide P7 was shown to stimulate splenocytes in a considerably lower capacity than the hybrid peptide, these results suggest that the significant T cell stimulatory effect of the epitope MPIWKFPDEEG is as a result of fusion of the single peptide P7 to the adjacent part of the hybrid peptide. In addition, detection of the MPIWKFPDEEG epitope which resides in the single peptide P7 may also suggest the specificity of the generated CD4 T cells in recognizing Her-2/neu. These results together signify the antigen specificity of our CRM-P467 as a novel conjugated peptide, comprising the capacity of not only inducing antibody responses by retaining the B cell epitopes of the single peptides P4, P6 and P7, but also stimulating T cell responses as a result of fusing the single peptides. Therefore, the high antibody responses as a consequence of proper T cell help seem to be triggered with the hybrid peptide as well as via bystander help of CRM-induced CD4 T cells.

Selection of appropriate epitopes and the use of a potent adjuvant system have both been proposed to be necessary for successful vaccination and generation of Her-2/neu immunity (3). Furthermore, in patients with advanced cancer a shift from Th1 to Th2 impairing the cell-mediated immunity has been reported [21], while lower recurrence rate and a longer disease-free survival was seen in patients with Th1-dominant responses [22]. Alum is a Th2-driving adjuvant [23] and based on its ability to enhance antibody production is widely used in human vaccinations against vaccine-preventable diseases, while Montanide is an emulsion adjuvant and capable of inducing both humoral and cellular responses and is increasingly used in recent cancer vaccine candidates [24]. In this study we therefore compared the immunogenicity of P467-CRM administered with either Alum or Montanide in Balb/C mice which have also been used in our previous preclinical studies evaluating the immunogenicity of the peptide vaccine [6].

Although both adjuvants were shown to further increase the antibody responses when administered with P467-CRM, the specific IgG, IgG1 and IgG2a antibodies against P467 hybrid peptide as well as the recombinant extracellular Her-2/neu were significantly higher in all mice groups immunized with Montanide (in all examined doses) compared to Alum. Our results also clearly showed that already with the lowest tested concentration (10 μg per dose) of the P467-CRM conjugate plus Montanide, the antibody titers were higher than compared with the highest tested concentration (50 μg per dose) of the formulation containing Alum, and indicated the superiority of Montanide- over Alum-containing formulation in eliciting IgG and IgG1 responses at any examined dose. The fact that already with low concentrations of the hybrid plus Montanide high immune responses are induced, is beneficial in terms of the manufacturing process of the formulation, and further signifies the advantage of Montanide in our CRM-conjugate hybrid peptide formulation. In addition, our results showed that even six months after the last immunization the serum IgG responses were higher in the groups of mice administered with Montanide than with Alum. Although comparing the decline rates of the responses showed no significant difference between the groups immunized with Montanide or Alum, our results suggest the potential of Montanide rather than Alum in inducing Th1-polarized and long term humoral responses. Similar results of higher antibody titers induced by P467-CRM-Montanide formulation, compared with P467-CRM-Alum, were observed both by ELISA assay using the recombinant Her-2/neu as the coating antigen as well as by FACS analysis examining the binding of the sera to native Her2/neu overexpressed on SK-BR-3 cell line, indicating the capacity of the strongly induced antibodies by P467-CRM-Montanide in specifically binding also native Her-2/neu. Examining the biological activity of anti-P467 IgG antibodies in inhibiting the proliferation of Her-2-overexpressing cell line SK-BR-3, our results also indicate that the induced antibodies possess specific anti-tumor properties and when combined with Herceptin® the inhibitory capacity is significantly higher than by Herceptin® alone suggesting polyclonal and additive anti-proliferative antibody responses against Her-2/neu by our hybrid peptide P467.

As mentioned, for successful tumor prevention the polarization of the immune response towards the Th1 type is highly important [3]. The Th1-produced cytokines, primarily IFNγ and IL-2 are essential for induction of cellular immunity, whereas Th2-produced cytokines like IL-4, IL-5 and IL-10 play a key role in eliciting humoral responses [25, 26]. The effectiveness of Th1 cells in the generation and maintenance of strong immunological memory has been shown [27], as well as association between secretion of IFNγ by CD4 T cells and their anti-tumor effects, e.g. by reducing proliferation and angiogenesis and enhancing apoptosis of tumor cells [28]. Further, effectiveness of IL-12 in inducing antitumor immunity in vivo through the activation of Th1 immunity has been demonstrated [26]; in agreement with this, we have earlier shown increased efficacy of our multi-peptide vaccine in reducing tumor growth in Her-2/neu transgenic mice when immunized together with IL-12, resulting in increased INFγ production and supporting the attribution of Th1 biased immune response to anti-tumor efficacy [7]. In the current study we have shown that upon re-stimulation with either CRM or P467, secretion of both IL-2 and IFNγ by splenocytes of mice immunized with P467-CRM plus Montanide was higher compared to Alum-containing formulation. Re-stimulation with the hybrid peptide P467 also induced IL-5 production- preferably in Montanide immunized mice, however in lower amounts than IL-2 and IFNγ. These results clearly indicate a Th1-poloarized immune response induced by the conjugated hybrid peptide administered together with Montanide. Similar results of IFNγ-associated T cell responses were also reported by Kenter et al. [29] when peptides of the E protein from human papillomaviruses (HPV16) were examined in conjugation with Montanide in a phase I trial.

A significantly higher number of IFNγ-producing CD8 T cells was observed in mice immunized with 25 μg peptide-CRM plus Montanide compared to mice administered with Alum. However it is unknown whether these cells are peptide or carrier specific, suggesting the possibilities that Montanide induces more efficient cross-presentation of peptide or carrier on MHC-I to CD8 T cells or that bystander activation of CD8 T cells occurs. Previous studies have shown that the activation of the desired IFNγ-producing CD8 T cells was as a result of bystander activation [30–32], suggesting the Montanide-driven bystander activation of the IFNγ-producing CD8 T cells also in our study.

As an oil-based adjuvant, Montanide persists at subcutaneous injection sites and formation of a depot effect is believed to contribute to immune enhancement of the adjuvant by slow release of the antigen associated with the adjuvant. Inflammatory and lymphocyte-trapping capacity of the adjuvant, by stimulating recruitment of antigen presenting cells and accumulation of lymphocytes in draining lymph nodes, respectively, are also involved in the immune enhancing effect of Montanide [18]. The Montanide used in our study, i.e. ISA-5-VG, contains vegetable-grade (VG) oleic acid derived from olive oil, and it has recently been used in a phase-I clinical trial enrolling patients with survivin-expressing solid tumor [33]. The study has shown that all subjects experienced one or more treatment-emergent adverse effects of general disorders, administration site conditions or gastrointestinal disorders, however less frequent adverse effects were observed in the subjects receiving the lowest dose (30 μg) and in overall the trial treatment was well tolerated with local injection-site reaction being the most frequent adverse effect [33]. In an additional phase 1/2a study, enrolling patients with advanced human papillomavirus-associated cancers and using the ISA-51VG adjuvant, mild injection site reactions were found to be related to the study treatment while all reported serious adverse events were unrelated to the study treatment [34].

## Conclusions

The results of this study show several advantages of the evaluated CRM-conjugated hybrid peptide over the previous multi-peptide vaccine with B cell epitope peptides individually conjugated to virosomes. Our novel CRM-conjugated construct not only retains the B cell epitopes of the single peptides with the capacity to induce antibody responses, the fusion of the single peptides has further generated T cell epitopes in the hybrid peptide capable of inducing cellular responses. When conjugated to CRM, our selected hybrid peptide induces considerably higher and more rapid immune responses compared to the virosome-conjugated peptides, elicits long-lasting antibody responses and exhibits antitumor activity. Also administration of the CRM conjugate with Montanide, compared to Alum, induces not only CD4 Th1-driven responses but also IFNγ-producing CD8 T cells already in the lowest concentration of the dose. Taken together, these results suggest the potential of our novel formulation containing CRM-P467 plus Montanide as a vaccine against Her-2/neu with polyclonal anti-tumor effect. The formulation P467-CRM-Montanide has recently been examined in a toxicology study and shown no signs of toxicity or inflammation when evaluated in dogs and rats (data not shown). A dose-escalating study, starting with 10 μg of the formulation, is being planned to be evaluated in a phase-Ib/II clinical trial enrolling Her-2/neu overexpressing cancer patients.

## Abbreviations

CRM: Cross reacting materials
